# A Proposed Clinical Decision Support Architecture Capable of Supporting Whole Genome Sequence Information

**DOI:** 10.3390/jpm4020176

**Published:** 2014-04-04

**Authors:** Brandon M. Welch, Salvador Rodriguez Loya, Karen Eilbeck, Kensaku Kawamoto

**Affiliations:** 1 Program in Personalized Health Care, University of Utah, 15 North 2030 East, EIHG Room 2110, Salt Lake City, UT 84112, USA; 2 Department of Biomedical Informatics, University of Utah, 26 South 2000 East, Room 5775 HSEB, Salt Lake City, UT 84112, USA; E-Mails: keilbeck@genetics.utah.edu (K.E.); kensaku.kawamoto@utah.edu (K.K.); 3 School of Engineering and Informatics, University of Sussex, Shawcross Building, Room Gc4, Falmer, Brighton, East Sussex, BN1 9QT, UK; E-Mail: s.rodriguez-loya@sussex.ac.uk

**Keywords:** clinical decision support systems, medical genetics, genomics, genetic testing, electronic health records, health information technology, personalized medicine, service-oriented architecture

## Abstract

Whole genome sequence (WGS) information may soon be widely available to help clinicians personalize the care and treatment of patients. However, considerable barriers exist, which may hinder the effective utilization of WGS information in a routine clinical care setting. Clinical decision support (CDS) offers a potential solution to overcome such barriers and to facilitate the effective use of WGS information in the clinic. However, genomic information is complex and will require significant considerations when developing CDS capabilities. As such, this manuscript lays out a conceptual framework for a CDS architecture designed to deliver WGS-guided CDS within the clinical workflow. To handle the complexity and breadth of WGS information, the proposed CDS framework leverages service-oriented capabilities and orchestrates the interaction of several independently-managed components. These independently-managed components include the genome variant knowledge base, the genome database, the CDS knowledge base, a CDS controller and the electronic health record (EHR). A key design feature is that genome data can be stored separately from the EHR. This paper describes in detail: (1) each component of the architecture; (2) the interaction of the components; and (3) how the architecture attempts to overcome the challenges associated with WGS information. We believe that service-oriented CDS capabilities will be essential to using WGS information for personalized medicine.

## 1. Introduction

The use of whole genome sequence (WGS) information for routine clinical care will greatly enhance the possibilities of personalized medicine, which include: (1) improving diagnostic accuracy and disease characterization; (2) targeting therapies to individuals; (3) identifying and preventing disease among high-risk individuals; (4) improving healthcare efficiency; and (5) reducing unnecessary costs [1,2]. With genomic information readily available to clinicians at the point of care, many of these goals can be realized. Indeed, significant investment has been made to improve genome sequencing technology and to reduce sequencing costs, making it easier to obtain a patient’s WGS for clinical care [3]. As a result, WGS information is now being used in the clinical setting for rare, undiagnosed disorders [4,5,6,7]. If current trends continue, it is anticipated that WGS information will soon be available for routine clinical care, thus enabling personalized medicine on a widespread scale [8].

While this is an intriguing prospect for patients, clinicians and researchers, significant barriers exist, which may hinder the effective use of WGS information in a routine clinical care setting. These barriers include: (1) static laboratory reports intended for human consumption; (2) the complexity of genetic analysis; (3) limited physician proficiency in genetics; and (4) the lack of genetics professionals in the clinical workforce [9]. These barriers, if not overcome, will likely hinder the ability of clinicians to provide personalized medicine using WGS information. Although there may be several approaches to overcome these barriers, we believe clinical decision support (CDS) provided within the clinical workflow provides the greatest opportunity to enable the effective use of WGS information in a routine clinical setting [9,10].

CDS entails providing clinicians, patients and other healthcare stakeholders with pertinent knowledge and/or person-specific information, intelligently filtered or presented at appropriate times, to enhance health and healthcare [11]. Examples of CDS include medication dosing support, order facilitators, point of care alerts and reminders, relevant information display, expert systems and workflow support [12]. Research on CDS has been conducted for several decades, with the established literature defining the features that contribute to successful CDS interventions [13,14]. To be effective, it is essential that CDS for WGS information follow these proven CDS practices and approaches; in particular, the integration of CDS with the clinician’s electronic health record (EHR) [9].

### 1.1. State of the Art

While CDS research is a well-established field, research on CDS for genetically-guided personalized medicine is a much younger, but growing, field. In a systematic review of CDS interventions for genetically-guided personalized medicine, Welch and Kawamoto identified 16 primary research articles describing CDS interventions using genetic information between 1990 to 2011 [15]. The majority of these CDS interventions tended to be stand-alone applications, which required re-entry of a patient’s clinical and genomic data by a clinician. Furthermore, these applications were largely limited to a single, or limited number, of genes (e.g., *BRCA1* and *BRCA2*) [16]. Recently, Tarczy-Hornoch *et al.* conducted a review of clinical reporting approaches for WGS (and whole exome) information in the EHR, which are currently implemented at six healthcare organizations [17]. These healthcare organizations developed, implemented and managed various approaches to EHR integration and CDS. However, the majority of these approaches were limited to static portable document format (PDF) reports (similar to pathology reports), and only two organizations leveraged the active CDS capabilities of the local EHR. The authors acknowledge that active CDS will be necessary for WGS information and that more sophisticated informatics tools will be necessary to scale up to meet the challenges of WGS information [17]. A more detailed description of these CDS examples and how they compare to the work described in this manuscript can be found in the Discussion section (see Section 4.1). In general, the literature on CDS for WGS information is still in its infancy [18].

### 1.2. Technical Desiderata

Given the critical role health IT will play in overcoming the barriers of WGS information and the specific challenges inherent in using genomic information, Masys *et al.* developed a technical desiderata for the integration of genomic information with an EHR [19]. These requirements, which were developed by a panel of experts, illustrate important considerations that should be addressed when developing health IT applications capable of supporting genomic information (see Table 1). Indeed, these desiderata are intended to overcome many of the barriers and challenges (also described in the ref. [19]) of using genomic information for clinical care.

**Table 1:** Genome-electronic health record (EHR) technical desiderata (Masys *et al*. [19]) for the integration of genomic data into electronic health records.

Desiderata Number	Desiderata Description
1	Maintain a separation of primary molecular observations from the clinical interpretations of those data
2	Support lossless data compression from primary molecular observations to clinically manageable subsets
3	Maintain the linkage of molecular observations to the laboratory methods used to generate them
4	Support a compact representation of clinically actionable subsets for optimal performance
5	Simultaneously support human-viewable formats and machine-readable formats in order to facilitate the implementation of decision support rules
6	Anticipate fundamental changes in the understanding of human molecular variation
7	Support both individual clinical care and discovery science

While the Masys desiderata provide a strong framework for integrating genomic data with the EHR, additional requirements are desirable for the integration of genomic information with CDS. Indeed, we believe it will be essential that genomic data are not only available within the EHR, but provided in a way that is useful to clinicians through CDS [9]. To address this need, Welch *et al.* developed an additional desiderata, to augment the Masys desiderata, specifically focused on the integration of genomic information with CDS (see Table 2) [20]. This work also describes the barriers and challenges that these additional requirements attempt to address.

**Table 2:** Genome-clinical decision support (CDS) technical desiderata (Welch *et al.* [20]) for the integration of genomic data with clinical decision support.

Desiderata Number	Desiderata Description
8	CDS knowledge must have the potential to incorporate multiple genes and clinical information
9	Keep CDS knowledge separate from variant classification
10	CDS knowledge must have the capacity to support multiple EHR platforms with various data representations with minimal modification
11	Support a large number of gene variants, while simplifying the CDS knowledge to the extent possible
12	Leverage current and developing CDS and genomics infrastructure and standards
13	Support a CDS knowledge base deployed at and developed by multiple independent organizations
14	Access and transmit only the genomic information necessary for CDS

These additional desiderata, when used together with the Masys desiderata, can provide a foundation to guide research and development on CDS for WGS information. As there are many barriers inherent in leveraging WGS information for CDS [9], incorporating these desiderata into the design and development process may help system developers overcome the challenges of using WGS information.

### 1.3. Study Objective

Given the importance that CDS will play in realizing personalized medicine through WGS information and the early stage of research and development in this domain [15,17,18], we put forth a theoretical CDS architecture based upon the technical desiderata and approaches utilized in prior work [15,17]. Indeed, this manuscript lays out the conceptual design of a proposed architecture and describes how each component of the architecture attempts to meet the requirements described in the technical desiderata. It is our intent to put forward this proposed architecture as a foundational reference for research and development on CDS for WGS information in the future.

## 2. Methods

We have leveraged our collective experience in the domains of genetics, bioinformatics and clinical informatics to propose a CDS architecture capable of supporting WGS information at the point of care. This manuscript, while describing the need for a particular approach or components, does not attempt to define the architecture components in sufficient detail necessary for implementation. Rather, this manuscript provides a business case and justification for the approaches and components used in this architecture.

### 2.1. Architecture Overview

Given the complexity of WGS information, the success of CDS in the genomic age will likely require an architecture that separates key capabilities into independently managed component parts [21]. As such, we advocate the use of a service-oriented architecture (SOA) as a design principle for our proposed CDS architecture. SOA is a software design methodology based on the interaction of separate, independent software components, known as services [22]. A service is a self-contained component that has well-defined, understood capabilities. SOA supports the reusability and standardization of processes, allowing for independent evolution and modifications to a particular service, reducing the burden of change on the overall system [23]. Because of the vast number of disparate health IT systems, the application of SOA principles offers several benefits to healthcare [24]. Indeed, research and development on SOA for CDS has led to several health IT standards and applications [25,26,27,28]. Furthermore, SOA-based CDS is currently under consideration for EHR certification criteria related to Stage III Meaningful Use guidelines [29].

#### SOA CDS for WGS Information

While SOA offers many benefits to health IT and CDS, we believe it will be necessary for WGS-enabled CDS [21]. Indeed, SOA can provide the agility needed to keep up with the rapidly evolving genomics knowledge base [30]. Furthermore, SOA allows for the scalability that is needed to handle the breadth of genomic applications in healthcare, particularly across multiple independent healthcare organizations [31]. In contrast, were a healthcare organization to develop and maintain their own CDS knowledge for WGS information, they could become overwhelmed by the time and cost of creating, managing and updating the CDS knowledge base for the entire genome [32]. This would be particularly challenging for the majority of healthcare organizations that have a limited clinical genomics presence [33]. Indeed, we believe it would be prudent to separate key components into independently managed services, which can be optimally maintained by third-party organizations.

**Figure 1:**
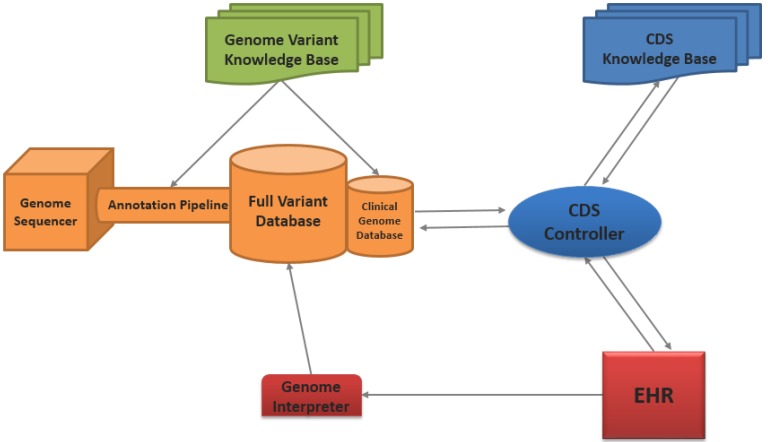
The proposed service-oriented architecture (SOA) architecture for whole genome sequence (WGS)-enabled CDS.

An SOA-based CDS architecture for the WGS information is an extension of previous efforts on SOA-based CDS in general [28] and early examples of CDS for genetic information [34,35,36]. The services and components required in our proposed architecture consist of genome sequencing and annotation, genome databases, genome variant knowledge bases, a CDS knowledge base, a CDS controller and the EHR (see Figure 1). A glossary of terms and brief descriptions are available in Appendix A. While some of these services and components are already available, some will need to be developed or enhanced to support an SOA-based approach. In subsequent sections of this manuscript, we describe each component in further detail, how they interact with each other and the enhancements that may be necessary.

### 2.2. Genome Sequencing and Annotation Pipeline

The first step in the entire process is to obtain the patient’s genome sequence, for either the whole genome, the exome, a gene panel or a more targeted, smaller subset of the genome. For a whole genome sequence, when compared to a reference genome, there are roughly three million single nucleotide variants per comparison. Two file formats for representing a patient’s set of genome variants include the variant call format (VCF) and the genome variant format (GVF) [37,38]. Both formats are able to represent various sequence and structural variations in the genome, such as single nucleotide polymorphisms, indels and substitutions.

#### Genome Annotation

Once the variants in the genome have been identified, it is necessary to prioritize variants that may have relevant phenotypic impacts. There are several sequential steps to variant annotation, which is referred to as the “annotation pipeline”. Initially, this process identifies variants occurring within known or predicted genes, regulatory regions, protein coding sequences or splice sites. Variants that occur within genes are assessed for clinical impact using curated genome variant knowledge bases (see Section 2.3), such as the Human Genome Mutation Database (HGMD), Online Mendelian Inheritance in Man (OMIM), ClinVar and other locus-specific mutation databases [39,40,41,42]. Additionally, computational interpretation approaches, such as VAAST, SIFT and PolyPhen, can be employed to prioritize or predict variant pathogenicity based upon the impact on the gene’s translational product [43,44,45,46]. Finally, gene functions, links to external knowledge resources and other variant metadata can also be included. The annotation pipeline can be developed internally by the organization sequencing the patient’s genome or using a service provided by a private company specializing in genome annotation services [47,48,49]. Currently, the entire sequencing and annotation pipeline is typically managed by a pathology laboratory. However, as genome sequencing technology advances, some speculate that this process could occur in the clinic [50]. In such cases, the proposed CDS architecture could still support this approach, as long as this component interacts, in a similar way, with the other components of the architecture, namely the genome variant knowledge bases (see Section 2.3) and the genome database (see Section 2.4).

### 2.3. Genome Variant Knowledge Base

A key part of the genome annotation process is to identify genome variants and assign a clinical impact, if known. A genome variant knowledge base is a repository of known genome variants and associated clinical interpretations of that variant. During the annotation pipeline, genome variant knowledge bases are ascertained for pre-existing knowledge on variants. There are many types of genome variant knowledge bases, which include: (1) privately-controlled knowledge bases, such as the Human Gene Mutation Database (HGMD) [39]; (2) open access, locus-specific knowledge bases, such as those created using the Leiden Open Variation Database (LOVD) [42]; (3) proprietary knowledge bases, typically owned and managed by genetic testing laboratories, who maintain exclusive access [51]; and (4) publicly available, centrally-managed repositories, such as ClinVar [41]. Typically, when a new variant is discovered or new information about a known variant is made available, this information will be recorded in one or more of these knowledge bases. Furthermore, curators may monitor publications and reports in order to update a knowledge base accordingly.

ClinVar, which is a publicly available central resource managed by the National Library of Medicine, represents a model wherein genome knowledge bases and laboratories (described above) can upload their expertly curated knowledge into one location. Previously, genome annotators may have had to use several different genome variant knowledge bases and pay to access particular knowledge. Furthermore, with a participatory approach to genome variant annotation, ClinVar may become a more robust and extensive knowledge base than any single locus-specific or laboratory-managed knowledge bases. Open access, locus-specific knowledge bases tend to be curated and maintained on a volunteer basis, making the knowledge available limited. While laboratory-managed knowledge bases contain the best variant knowledge, they are also: (1) limited by the number of unique variants observed by that laboratory; and (2) may have tightly controlled access to the variant knowledge in order to maintain a competitive advantage over other testing laboratories [51]. Nevertheless, if ClinVar is embraced by the diagnostic laboratory community with the support of the ClinGen effort [52], the laboratory knowledge bases will likely serve as one of the most important sources of variant annotations.

#### 2.3.1. Variant Clinical Interpretations Categories

The clinical interpretation categories for sequence variations stored in the genome variant knowledge bases may follow recommendations set by the American College of Medical Genetics and Genomics (ACMG) and others [53,54]. The ACMG recommendations include classifications, such as “pathogenic”, “likely pathogenic”, “variant of unknown significance” (VUS), “likely benign” and “benign” for diseases caused by genes. Pharmacogenomics (PGx) classification categories include “ultrarapid metabolizer”, “intermediate metabolizer” and “poor metabolizer” for genes impacting drug metabolism [55]. Some have also used allele classifications (e.g., *1/*2) to represent PGx variants for CDS, though this practice is becoming increasingly complicated as more variants are discovered [56]. Unfortunately, many of these classification categories are not used consistently, and many labs create their own classification categories, which may result in interpretation discrepancies and confusion among clinicians. Therefore, in order to facilitate the widespread adoption of CDS, future effort may be necessary to promote standardized variant classification definitions [57].

#### 2.3.2. Variant Knowledge Management

Our understanding of genomics in health is still relatively nascent. However, as research into the human genome grows, so too will the understanding of the health impacts of genome variants. This growth in understanding will likely lead to frequent and significant changes to variant classifications. To illustrate, over a seven year period, the Partners HealthCare Center for Personalized Genetic Medicine’s Laboratory for Molecular Medicine genome variant knowledge base, managed by the GeneInsight Suite, reclassified nearly 15% of their original classifications, with almost one third of those initially being VUS [30]. As such, genome variant knowledge bases will play an important role in independently managing the clinical interpretations of variants for the genomic CDS architecture. Not only can the most up-to-date variant classification be available during the annotation process, but if a clinical interpretation of a variant later changes, the variant classification for a particular patient’s genome can be automatically updated (see Section 2.4). In such cases, changes in clinical interpretations will likely need to be versioned and tracked to account for potential liability concerns. Nevertheless, this separation of concerns through the SOA allows CDS to use the most up-to-date variant knowledge, while being free of dependencies that are timely and costly to update.

### 2.4. Genome Databases

The storage of a patient’s annotated variants is central to the proposed CDS architecture. With a patient’s genome stored and accessible, a patient’s genetic information can be available for CDS when needed.

#### 2.4.1. Genome Data Considerations

Although the size of a genome dataset can be significantly reduced using variant file formats, much of the resulting data may still be unnecessary for most CDS use cases [9]. For example, these genome variant files contain a comprehensive set of all variants in the patient’s genome, whether or not they are associated with a known gene or phenotype. As such, it may be unnecessary to make all variants available for CDS, particularly those which have no known association with genes and or phenotypic impact. Furthermore, while genome sequence metadata and annotations are important for quality assurance, variant classification and versioning, some of this metadata may not be necessary for the purposes of CDS. Examples of this metadata include the reference sequence used, sequence coverage, population frequency and reference copy number.

These examples are important to consider when trying to simplify CDS knowledge to the extent possible. To illustrate, in cases where there are hundreds of known pathogenic variants within a particular gene, it may not be efficient to write CDS knowledge for every known variant, particularly when the clinical phenotypes of different variants are identical and variant clinical interpretations can change. In certain use cases, it may be sufficient to simply represent a variant by its clinical interpretations. For example, heterozygous mutations in the *MLH1* gene cause hereditary nonpolyposis colorectal cancer, therefore a simple CDS rule using this approach could be: “*If* [gene = “*MLH1*”] *has* [variant classification = “*pathogenic*”], *then* [recommendation = “*recommend colonoscopy to patient*”].” Nevertheless, for specific use cases where the variant location and effect (e.g., frameshift mutations) within a gene produces a unique phenotype or when a particular allelic variant is important for pharmacogenomic dosing, such information can still be made available to CDS when needed.

#### 2.4.2. Database Approach

As a result, for the purposes of CDS, we advocate the use of a clinical genome database consisting of only a patient’s clinically relevant variants and a full genome database consisting of all variants and genome metadata. The clinical genome database should consist primarily of variants in or near genome regions associated with phenotype (e.g., genes), with associated data elements required for CDS knowledge. Data elements and possible standards that could be used for CDS include: (1) genome type (e.g., germline or somatic); (2) gene name in the standard HUGO Gene Nomenclature Committee (HGNC) format [58]; (3) the variant in a standard format, such as the Human Gene Variation Society (HGVS) format [59]; and (4) the variant clinical classification, as provided by the genome variant knowledge base (see Section 2.2). Other potentially important elements that could be useful for CDS include genotype, haplotype, tissue type and genome copy number. However, it is currently unknown exactly which genomic information will be necessary for CDS; future research will help determine which information is important.

Other reasons for creating a simplified clinical genome database are to improve performance and security. In an SOA architecture with multiple independent components, speed and efficiency are a top priority, particularly for CDS. Reducing the need for a database query to filter through unneeded data is likely to improve performance, particularly when such databases grow to include genomes of many patients. Furthermore, limiting genetic information available to external queries promotes privacy and security, as clinically unnecessary genomic data could potentially be used to uniquely identify anonymous genomes [60].

Finally, while we describe the two databases as being separate, this can be a virtual separation or a physical separation. Nevertheless, there will need to remain a connection that will allow for changes in our understanding of the human genome. Indeed, data available in the full genome database will be available to the clinical genome database if and when it becomes clinically relevant and useful to CDS. Furthermore, just as a genome database is made available for clinical care, it should also be available for research, using many of the same service-based approaches [21].

### 2.5. The Roles of the Electronic Health Record

The EHR represents an important role in the proposed architecture, as it is responsible for collecting and storing the patient’s clinical data required for CDS. Furthermore, it provides the mechanism by which CDS interacts with the end-user at the point and time of care. Indeed, to be effective, CDS for WGS should be integrated within the EHR clinical workflow, similar to how other non-genomic CDS is provided. It will likely not be sufficient or desirable to have a stand-alone CDS application for WGS information.

#### 2.5.1. EHR as a Repository of Clinical Data

EHRs serve as the primary source of collecting and storing clinical information that will be used to provide CDS. While EHRs have traditionally functioned as clinical data repositories, most EHRs currently do not have an effective way of storing genetic information [61]. Furthermore, with competing higher-priority demands (e.g., Meaningful Use) among EHR vendors, this may not change in the near future. Therefore, our approach is to store genomic data separately from the clinical data in the EHR (see Section 2.4) and leverage service-based capabilities to obtain the clinical and genomic data required for CDS. This approach reduces the burden on EHR developers to build genome-specific capabilities, while allowing them to continue serving as the primary source of clinical data. Unfortunately, most EHRs use their own approaches for collecting and storing clinical data, which can be challenging for scalable CDS solutions [62]. However, this challenge can be overcome by mapping various data models to a common standardized data model, used specifically for CDS. A CDS data model being considered for EHR certification criteria related to Meaningful Use Stage 3 is the Health Level 7 Virtual Medical Record (vMR) standard [63].

#### 2.5.2. CDS Interface with End Users

In addition to collecting and storing clinical data, EHRs are also responsible for the triggering of a CDS request and then presenting the CDS results in an effective way to end users. CDS can be triggered in a variety of situations, such as: (1) when the patient’s record is opened or a certain EHR view is selected; (2) when a drug or procedure is ordered; (3) when clinical documentation occurs within the EHR; or (4) at a routine time interval. Furthermore, the EHR can present CDS results within the clinical workflow of the clinician [14]. To this end, CDS results can be displayed as point of care alerts or reminders, relevant information displays, care recommendations, order facilitators or workflow support [12,64]. In principle, all the same CDS capabilities, which are currently available within EHRs, should also be used to trigger and present CDS for WGS information according to the CDS best practices [13,14].

#### 2.5.3. Leveraging Available EHR Capabilities

To provide CDS for WGS information within the EHR, the proposed architecture should primarily rely on EHR capabilities that are currently supported or likely to be supported in the near future. To illustrate, the EHR market currently consists of hundreds of vendor solutions, each with their own development roadmaps and timelines [65]. Being reliant on custom EHR integration solutions may be an inadequate approach to attaining widespread and consistent use of CDS for WGS information [35]. Rather, aligning the proposed CDS architecture with current and potential future EHR capabilities, mandated by certification criteria, offers a pragmatic and effective solution. Of note, service-based CDS capabilities for EHRs are currently under consideration for Meaningful Use Stage 3 [25]. Moreover, some major EHR system vendors already support service-based CDS capabilities [66].

In summary, there are many advantages to leveraging EHR capabilities that are currently available and/or are aligned with relevant EHR certification criteria for WGS-driven CDS. As this approach is not dependent upon internal EHR development timelines and prioritization, it offers a greater chance of gaining widespread and consistent distribution across multiple EHR vendors and healthcare organizations. As such, this proposed architecture is designed to leverage existing EHR capabilities and align them with ongoing developments in health IT.

### 2.6. CDS Knowledge Base

CDS entails providing person-specific care recommendations or knowledge, which can be used to enhance health and healthcare [67]. CDS knowledge bases contain representations of clinical knowledge (CDS knowledge) in the form of logic, decision rules, expressions, guidelines and algorithms that support the provision of care, based upon a patient’s clinical and genomic information. In an SOA CDS architecture, the CDS knowledge base is encapsulated as an independent unit by a service. This service receives patient-specific information provided by the CDS requester, processes this information and returns a CDS result. As such, this approach reduces dependencies upon requesting EHR systems (requestor), if standardized data models and terminologies are used [68]. Furthermore, as the CDS knowledge is agnostic to how or where the data is originally stored, its primary concern is to process the standardized patient data according to the knowledge it contains.

Likewise, as CDS knowledge authoring and maintenance can be time consuming, keeping the maintenance of variant classifications separate (see Section 2.3) from CDS knowledge will promote efficiency in CDS knowledge management. This approach allows variant classifications to freely change without needing to update CDS knowledge bases, as well. Finally, as CDS knowledge could become complicated for genomic information, simplifying the knowledge to the extent possible is a desirable attribute. As described in Section 2.4, this can be achieved by writing CDS knowledge using a gene and an associated clinical interpretation. Creating CDS knowledge for every possible variant within a particular gene, for which there are thousands of variants known and potentially many more unknown, will be inefficient [9].

#### CDS Knowledge Development and Management

As a result of the SOA approach, CDS knowledge bases can be deployed and maintained by an independent entity specializing in the development and management of CDS knowledge. For example, an entity that specializes in developing and optimizing pharmacogenomic dosing regimens can deploy their knowledge as a service-based CDS knowledge base, allowing subscribing organizations to leverage the most up-to-date knowledge, provided by that entity [69]. Likewise, medical societies, which develop disease-specific care guidelines and recommendations, can deploy their work as a CDS knowledge base and allow member institutions to utilize the care guidelines and recommendations in the form of CDS [70]. Furthermore, the ability to leverage independently developed CDS knowledge could increase a healthcare organization’s access to CDS capabilities and promote competition among CDS knowledge authors. Similarly, this SOA approach also supports the ability to share the same CDS knowledge among many healthcare organizations. This is important, because it is unlikely that a single healthcare organization will be able to maintain all its own CDS knowledge for WGS information, particularly for small and rural healthcare organizations with limited genomics expertise [9].

### 2.7. CDS Controller

As previously described, genomic data required by the CDS knowledge base will not be stored with the clinical data from the EHR (see Section 2.5). Rather, a patient’s genomic information will be stored and maintained in a separate genome database (see Section 2.4) [21]. With the separation of clinical data from genomic data, as proposed in this CDS architecture, a component that links and coordinates the other components of the architecture together will be required. Indeed, this is the primary role of the CDS controller, which is to combine clinical data from the EHR with genetic data from the clinical genome database into a complete data package for the CDS knowledge base. The CDS controller compares the received patient data to the CDS knowledge data requirements, which can include required data elements and desirable formats. The CDS controller can also facilitate workflow-appropriate triggering, perform terminology mappings, exclude unneeded clinical data, request additional data from other sources and enable end-user interaction, as necessary [26]. The functions of a CDS controller, in our proposed CDS architecture, consists of the following sequential steps:

1. The CDS controller obtains clinical data from the EHR in a standardized format (e.g., vMR), as a result of a CDS trigger within the EHR.
2. The patient data is compared with the data requirements for the requested CDS knowledge module. In the case of our architecture, the CDS controller will identify that the patient’s genomic information required by CDS knowledge is missing and will make a request to the genome database for that information.
3. The CDS controller obtains the patient’s genomic information from the clinical genome database, as specified by the CDS knowledge data requirements.
4. The CDS controller then merges the patient’s genome information with the clinical information into a single vMR file.
5. The complete data package is subsequently transmitted to the CDS knowledge base for evaluation.
6. After CDS evaluation, the CDS controller receives the CDS response from the CDS knowledge base. At this point, the CDS controller can then process the CDS responses with additional workflow requirements (e.g., human review and approval of CDS recommendation), if necessary.
7. The CDS response is relayed to the EHR for end-user presentation.

While the CDS controller is described as being a separate component in this architecture, it is certainly feasible for the CDS controller to be an embedded function within an EHR. Indeed, such a scenario is described in another manuscript [28].

### 2.8. Genome Interpreter

The genome interpreter, while not directly involved with CDS as described above, may be an important component to clinicians who desire to manually review variants in a patient’s genome. As CDS may not be able to represent every possible clinical scenario, the capacity to manually review variants, clinical impact and relevant metadata about a patient’s genome will be important. Examples of genome interpreters include those provided by commercial genome annotation companies [47,48,49]. While these solutions are available as stand-alone applications, ideally, they should be made available to clinicians within their EHR.

## 3. Results

### Meeting the Technical Desiderata

An objective of the proposed CDS architecture for WGS information is to satisfy the requirements in the technical desiderata [19,20]. Table 3 represents a summary of barriers to using WGS information in the EHR and CDS, the desiderata requirements that are designed to address the barrier and a description of how our proposed architecture attempts to satisfy each requirement in order to overcome the barrier.

## 4. Discussion

### 4.1. Comparison of Proposed Architecture to Prior Work on CDS for Genomics

As described in the Introduction, there is a growing research base on CDS interventions for genomics [17]. Indeed, the design and capabilities of many of these CDS examples provide the basis for the conceptual approaches described in our proposed architecture. As described earlier, of the organizations described in the Tarczy-Hornoch *et al.* review [17], all organizations developed and managed their own genome annotation process, each developed custom genome variant knowledge bases and most had primitive CDS capabilities, primarily limited to PDF reports. Furthermore, report generation was dependent upon local experts unique at each institution, an approach that is unlikely to be scalable.

As a noteworthy example, the GeneInsight Suite is a stand-alone, web-based interface designed to manage and communicate genome variants and clinical interpretations between clinicians and laboratories [30,34,71]. The GeneInsight Suite is an example of an application that can support a genome variant knowledge base managed by a laboratory and maintain current clinical interpretations in a genome database. While this application focuses on managing variant knowledge and communicating updates to clinician end-users, the knowledge communicated is largely limited to the patient’s genomic information, variant clinical interpretation and a generic variant report. Furthermore, as the application currently exists separately from the EHR, its ability to leverage clinical data and provide patient-specific CDS based on clinical and genomic data within the EHR workflow is limited [9]. Indeed, tighter integration with the EHR and CDS is an important future effort acknowledged by the developers of the GeneInsight application [30].

**Table 3:** A summary of how the proposed CDS architecture satisfies the EHR and CDS WGS desiderata.

WGS barriers	Desiderata requirements	How the proposed architecture addresses requirements
Clinical interpretations of genomic information can be dynamic [30]	(Desiderata #1) Maintain a separation of primary molecular observations from the clinical interpretations of those data	The genome variant knowledge bases exist separately and independently from the genome databases
WGS information contains a large amount of redundant and non-relevant data [38]	(Desiderata #2) Support lossless data compression from primary molecular observations to clinically manageable subsets	Genome variant file formats are based on a reference sequence, and a clinical genome database is used
Genomic results may be different based upon laboratory methods [72]	(Desiderata #3) Maintain a linkage of molecular observations to the laboratory methods used to generate them	Laboratory methods are included with the variant file in the full genome database
A majority of a patient’s 3,000,000+ genome variants will not have a clinical impact [4]	(Desiderata #4) Support the compact representation of clinically actionable subsets for optimal performance	Compact representation of clinically actionable informatics are available in the clinical genome database
Computing on the genome will require data representations that are hard for humans to understand [61]	(Desiderata #5) Simultaneously support human-viewable formats and machine-readable formats in order to facilitate the implementation of decision support rules	The machine-readable data format is used throughout the architecture, whereas a human viewable format is available through the genome interpreter
Our understanding of the human genome is nascent and may change significantly in the future [73]	(Desiderata #6) Anticipate fundamental changes in the understanding of human molecular variation	The proposed SOA architecture design allows for the flexibility of components to adapt to additional requirements as needed
Using available clinical and genomic information will be essential for research and discovery [74]	(Desiderata #7) Support both individual clinical care and discovery science	The same methods used to gather clinical and genomic data for CDS can be used for research, as well
Relatively few diseases are caused by a single genetic variant alone [75]	(Desiderata #8) CDS knowledge must have the potential to incorporate multiple genes and clinical information	The CDS controller is able to collect all required clinical and genomic data required by the CDS knowledge base
CDS knowledge may evolve independent of variant classifications [30]	(Desiderata #9) Keep CDS knowledge separate from variant classification	The CDS knowledge base is a separate component from the genome variant knowledge base
Many organizations, with various EHR platforms, will likely not be able to develop their own CDS for WGS information [65]	(Desiderata #10) CDS knowledge must have the capacity to support multiple EHR platforms with various data representations with minimal modification	The architecture uses industry standards and approaches for scalable, interoperable CDS that are being considered for inclusion in EHR certification criteria related to Meaningful Use Stage 3
A single gene can have 100s–1,000s of variants with various clinical impacts [76]	(Desiderata #11) Support a large number of gene variants, while simplifying the CDS knowledge to the extent possible	The information in the clinical genome database and required for CDS can simply consist of the gene and its clinical interpretation
Re-inventing prior standards work on genomics and CDS just for this use case may prove to be futile [57]	(Desiderata #12) Leverage current and developing CDS and genomics infrastructure and standards	Health IT and genetics standards are used throughout the architecture
No single entity will be able to develop and maintain all possible CDS knowledge for WGS [69]	(Desiderata #13) Support *a CDS knowledge base* deployed at and developed by multiple independent organizations	Service-based CDS supports CDS knowledge developed and maintained by multiple, independent organizations
The file size and security concerns for WGS information are important [77]	(Desiderata #14) Access and transmit only the genomic information necessary for CDS	The CDS controller requests only the genome data needed for CDS knowledge

Furthermore, several groups have implemented preemptive pharmacogenomics (PGx) CDS within EHRs, namely the PREDICT project at Vanderbilt in Nashville, Tennessee [35,78]; a group at St. Jude Children’s Research Hospital in Memphis, Tennessee [79,80]; and the CLIPMERGE project at Mt. Sinai Hospital in New York City [36]. The PREDICT project provides an example of active CDS for genotype information that is integrated within the clinical workflow of the EHR. Indeed, this CDS capability for PGx is built into the order entry component of Vanderbilt’s homegrown EHR system. Furthermore, all genotype results for a patient are stored in a database repository, separate from the EHR, with actionable genotype results and their interpretations stored as a laboratory result within the EHR. As the CDS for this project was developed and built into the EHR by an internal panel of experts, its scalability is limited beyond their own institution. Furthermore, the CDS rules do not incorporate clinically relevant non-genomic information into the decision process [35,78]. St. Jude Children’s Hospital also takes an approach of storing genetic test results directly in the EHR (Cerner). The EHR uses its native CDS capabilities to provide alerts and recommendations, which are developed and maintained by the institution. Again, this approach will likely be challenging to scale beyond their institution and beyond the PGx use case. Finally, for the CLIPMERGE project, actionable PGx genetic test results derived from the institution’s research bio-bank (BioMe) are combined with relevant clinical information extracted from the institution’s EHR (Epic) in an external CLIPMERGE database. An external CDS rules engine also processes the patient data from the database and returns the results back to the EHR in real time. The CLIPMERGE approach uses a separation of components and is likely the closest example to the approach described in the current manuscript. However, with clinical data extracted from the EHR and stored in a separate database along with the genetic information, it is also unclear if this approach could support WGS information and whether it can be easily scalable [57].

#### Originality and Uniqueness of the Proposed Architecture

In summary, these examples represent important contributions to CDS approaches for genomics. While not all these solutions are designed for WGS information and some of these approaches would struggle to support WGS information, they contain important design approaches that can be implemented in a scalable architecture, able to support WGS information. Indeed, many of the design principles in these examples were a source of inspiration and adopted for our proposed CDS architecture. Indeed, we believe it will require the coordination of several of these proven components to build a CDS architecture capable of effectively leveraging WGS information. As a result, we have proposed an architecture, which uses many of these proven design approaches, that is able to provide CDS for WGS information on a widespread scale. We believe that our proposed architecture approach, described in this manuscript, will be important for achieving this goal.

### 4.2. Barriers Still to Overcome

While the proposed architecture aims to overcome many barriers related to genetic information, there are still many barriers to overcome before this architecture can be realized on a widespread scale. For instance, our understanding of the human genome and, thus, the annotation process is still in the relatively early stages. In fact, the reference genome used during the annotation process will likely change in the future. Likewise, many caveats, such as race and family health history, must be considered for an accurate clinical interpretation of a variant. Furthermore, as described in Section 2.3, many variant classification categories are used to describe the clinical impact of variants. As these classifications may be used when authoring CDS knowledge, it is important that a standard, well-defined variant classification system is consistently used to describe a variant’s clinical impact. While ClinVar has a set of classifications that are currently used [81], they are probably not sufficient to represent clinical impact with the specificity needed. Furthermore, with regards to ClinVar, there may be situations that arise involving differing expert interpretations for the same variant. In such cases, the various interpretations will have to be harmonized in some way by ClinVar or a related entity.

With regards to CDS infrastructure, service-based CDS capabilities are still in the early stages of industry adoption and, thus, still fairly limited with regards to technical capabilities, available standards and available CDS knowledge. Indeed, using the SOA CDS approach described in here will require that significant gaps in standards and technology be addressed. Furthermore, even with the technical capabilities in place, there are still many non-technical issues for service-based CDS that will need to be overcome, such as legal uncertainties regarding medical liability and questions regarding the financial sustainability of a services-based approach to CDS delivery. While such issues are important and must be addressed to enable services-based CDS for WGS information, these issues are also of interest to, and being addressed by, the larger CDS community. For instance, the consistent and widespread adoption of a service-based CDS architecture may be greatly enhanced by related EHR certification criteria that are under consideration for Meaningful Use Stage 3, due out in 2017 [82]. Indeed, efforts are currently underway in the Health eDecisions initiative (led by the manuscript co-author, Kensaku Kawamoto) to develop and pilot standards that are being considered for this purpose [83]. Of note, however, regulations and EHR certification criteria related to Meaningful Use Stage 3 are still under development and are subject to change. Nevertheless, some major EHRs have already implemented, or have plans to implement, service-based CDS capabilities in the near future, irrespective of Meaningful Use requirements.

### 4.3. Current Efforts and Future Direction

While this manuscript is largely theoretical, current efforts by the authors are underway to build and test a functional prototype of this system, with greater technical details regarding specifications. Indeed, this prototype currently follows the methods described in this paper in an attempt to meet the requirements in the technical desiderata. Once demonstrated with a prototype, it would be appropriate to build out a more robust infrastructure and implement the architecture on a small scale within a clinical setting. Such an implementation could begin with single gene test results and then move to more complex gene panels and whole genome sequences. Additionally, research and experience from these implementations may determine that performance issues and security (see Section 2.4) may be less of a concern than previously thought. Moreover, as the architecture capabilities become available to more healthcare providers, it will become appropriate to develop genome-specific CDS knowledge.

Furthermore, as mentioned in Section 2.4 on genome storage, future research will be needed to determine which genomic information will be essential for CDS knowledge. Indeed, a systematic review and analysis of potential CDS knowledge for genomic information could help determine the most important elements for genome-based CDS. Furthermore, the current architecture is primarily focused on: (1) simple kinds of genomic variation (e.g., SNP variants within genes); and (2) variants with known clinical impact. However, current and future genomic discoveries may uncover complex interactions, which may require additional architectural considerations and modifications in order to support CDS. Likewise, by incorporating variant prioritization algorithms, such as VAAST, CDS could also become more involved with the interpretation of novel variants [45,46]. As a result, we do not presume the currently proposed architecture to be the final solution for WGS-based CDS. Rather, the current architecture provides a foundation for future development and modifications as our understanding of the genome and health grows.

## 5. Conclusions

The availability of a patient’s whole genome sequence has the potential to facilitate the practice of personalized healthcare in the clinic. While research efforts are producing significant discoveries in support of personalized medicine, many barriers exists that limit the effective utilization of these discoveries in a clinical setting. Such barriers include the complexity of genomic information, the changing nature of the understanding of the genome, current result reporting methodologies and the limited availability of clinical genomics experts [9]. However, effectively designed CDS, provided within the clinical workflow, offers a potential solution to support the effective clinical utilization of WGS information. Indeed, a well-coordinated, service-based CDS architecture represents a practical solution to provide WGS-enabled CDS at the point of care.

## Glossary of Key Terms Used

CDS controller: A component of a SOA architecture which links several services together
CDS knowledge: A representation of clinical knowledge in the form of logic, rules, expressions, guidelines or algorithms
CDS knowledge base: A repository of CDS knowledge
Clinical genome database: A repository which stores only variants of known or potential clinical importance
Clinical interpretation: The clinical impact of a variant
Full genome database: A repository which stores all variants of an individual’s genome
Genome annotation: The process of locating and identifying key features of a genome
Genome interpreter: A visual interface which allows a clinician to manually review a patient’s genome variants
Genome sequencing: The process of obtaining the DNA sequence of an individual
Genome variant knowledge base: A repository of variants and associated clinical interpretation
Genome variant: A difference in a genome relative to a reference genome sequence
Service: A self-contained component with well-defined, understood capabilities
Service oriented architecture (SOA): A software design methodology which contains several independent services
